# Mechanistic regulation of HERV activation in tumors and implications for translational research in oncology

**DOI:** 10.3389/fcimb.2024.1358470

**Published:** 2024-02-06

**Authors:** Elena A. Cherkasova, Long Chen, Richard W. Childs

**Affiliations:** Laboratory of Transplantation Immunotherapy, National Heart Lung and Blood Institute, National Institutes of Health, Bethesda, MD, United States

**Keywords:** HERV expression, carcinogenesis, epigenetics, cancer antigens, cellular immunotherapy

## Abstract

Transcription of distinct loci of human endogenous retroviruses (HERVs) and in some cases, translation of these transcripts have been consistently observed in many types of cancer. It is still debated whether HERV activation serves as a trigger for carcinogenesis or rather occurs as a consequence of epigenetic alterations and other molecular sequelae that characterize cellular transformation. Here we review the known molecular and epigenetic mechanisms of HERV activation in cancer cells as well as its potential contribution to carcinogenesis. Further, we describe the use of HERV expression in cancer diagnostic and characterize the potential of HERV-derived antigens to serve as novel targets for cancer immunotherapy. We believe this review, which summarizes both what is known as well as unknown in this rapidly developing field, will boost interest in research on the therapeutic potential of targeting HERV elements in tumors and the impact of HERV activation in oncogenesis.

## Introduction

Progress in next-generation sequencing (NGS) technologies as well as development of new algorithms for genome analyses has provided a plethora of new data and has led to the rapid identification of and research on various types of noncoding RNA (ncRNAs), including human endogenous retroviruses (HERVs) (Lv et al., 2014; Chen and Li, 2019; Smith et al., 2019; Kolbe et al., 2020; Lu et al., 2021; Bonaventura et al., 2022). NcRNAs are transcribed in most regions of the human genome and divided into two major groups according to their size and processing: small ncRNAs (less than 200 nt in length) and long ncRNAs (longer than 200 nt), which includes HERVs. Multiple studies have demonstrated that ncRNAs formerly considered as “junk” relics due to their lack of coding potential can be actively involved in gene expression regulation via different variable mechanisms. Although current information on ncRNAs and their abundance in the human genome is still inadequate, it is now clear that they participate in many critical physiological and pathological processes, including carcinogenesis.

Human endogenous retroviruses, comprising at least 8% of our genome, are the remnants of ancient exogenous retroviruses that infected and incorporated into primates’ germ line over the past 100 million of years (Griffiths, 2001). Complete HERVs retain similar sequences to exogenous retroviruses and are composed of *gag, pro, pol*, and *env* regions flanked by two long terminal repeats (LTRs). Initially HERVs were thought to be inert genomic sequences, as most of them are replication defective because the open reading frames (ORFs) of retroviral genes have been evolutionary degraded by mutations and deleterious insertions or deletions to prevent production of viral particles. Furthermore, HERVs are subject to layers of control by epigenetic mechanisms that keep them silenced in adult human tissues. However, it is now known that HERV elements are transcriptionally and sometimes even translationally active in humans, mostly under pathological conditions that interact with host surveillance mechanisms. Expression of HERV-related genetic information and its involvement in human pathological processes are mostly unknown and under active investigation. There are numerous publications describing HERV activation in different types of cancer (addressed in this review), as well as in other diseases such as type 1 diabetes (Levet et al., 2017), neurological diseases such as multiple sclerosis (MS) (Perron et al., 2012a; Madeira et al., 2016; Hartung et al., 2022; Irfan et al., 2022) and amyotrophic lateral sclerosis (ALS) (Douville et al., 2011; Alfahad and Nath, 2013; Arru et al., 2021; Phan et al., 2021), and psychiatric diseases such as schizophrenia (Aftab et al., 2016; Perron et al., 2012b; Tamouza et al., 2021) and bipolar disorder (Perron et al., 2012b; Tamouza et al., 2021). Over the last few years, research in this field has led to growing interest in exploring HERV-originated products as biomarkers for disease and specific HERV-derived antigens as targets for immunotherapy.

The current literature on HERVs presents several different classifications and nomenclature that are sometimes ambiguous. The original nomenclature of HERVs was based on the first letter amino acid code of the tRNA of the primary binding site in reverse transcription (e.g. HERV-K for lysine, HERV-H for histidine, etc.). However, some HERV elements were initially described and named according to: (i) the particular amino acid motif, like HERV-FRD (Blaise et al., 2003), (ii) the original clone number, such as HERV-S71 (Haltmeier et al., 1995), (iii) the name of neighboring gene, like HERV-ADP (Jern et al., 2005), or the cancer type where the HERV element was discovered, such as CT-RCC HERV-E (Takahashi et al., 2008). Moreover, multiple genetic modifications, that occurred via recombination events over millions years of human genome evolution have resulted in a vast and variable number of HERV sequences. A comprehensive phylogenetic analysis performed with RetroTector software has led to HERVs being classified into three classes (e.g. Gamma-like, Beta-like and Spuma-like classes), 10 super groups and 39 canonical groups (Sperber et al., 2007; Vargiu et al., 2016). Further, a revised nomenclature of transcribed HERV loci (in the format ERV + group symbol + unique number) has been developed to sort HERVs into groups based on Repbase classifications (Mayer et al., 2011).

Descriptions of HERVs using classical virology or genetics are difficult as they are neither factual retroviruses nor do they encode physiological genes. The only exceptions are a limited number of “domesticated” HERV genes that have been discovered to contribute to normal tissue functions. In particular, protein products of two genes of the HERV-W family, namely HERV-W-originated Env glycoprotein Syncytin-1 and HERV-FRD Env glycoprotein Syncytin-2, have been implicated in the process of proper placentation and cell-cell fusion (Blaise et al., 2003; Esnault et al., 2008; Lavialle et al., 2013; Lokossou et al., 2014). Both of them function to fuse the syncytiotrophoblast layer and villous cytotrophoblasts, contributing to the stability and constant renewal of the placenta (Denner, 2016). Intriguingly, another Env protein called Suppressyn, which is encoded by HERV-Fb1 and shares the same ASCT receptor (Sugimoto et al., 2013), has been recognized as a Syncytin-1-specific inhibitor. Additionally, Suppressyn might serve as a mediator in syncytialization regulation to prevent aberrant placentation (Sugimoto et al., 2019).

Among the HERVs, the HERV-K group is the most recently integrated into our genome and therefore generally preserves full-length ORFs encoding Gag, Pro, Pol and Env proteins which are needed for viral assembly (Hohn et al., 2013). Interestingly, HERV-K proteins and viral-like particles were detected in human blastocysts, suggesting HERV-K may be involved in normal human embryogenesis at early stages, albeit more research is needed to substantiate this finding (Grow et al., 2015). Recently, Liu et al. found elevated levels of HERV-K mRNA and proteins along with an accumulation of HERV-K retrovirus-like particles in human mesenchymal progenitor cells (hMPCs) with premature aging phenotypes (Liu et al., 2023). Intriguingly, HERV-K resurrection in a cellular senescence model was found to be consistent with aging-related epigenetic derepression marks. This study provided first insight into possible role of HERV products in cellular senescence mechanisms.

**Figure 1:**
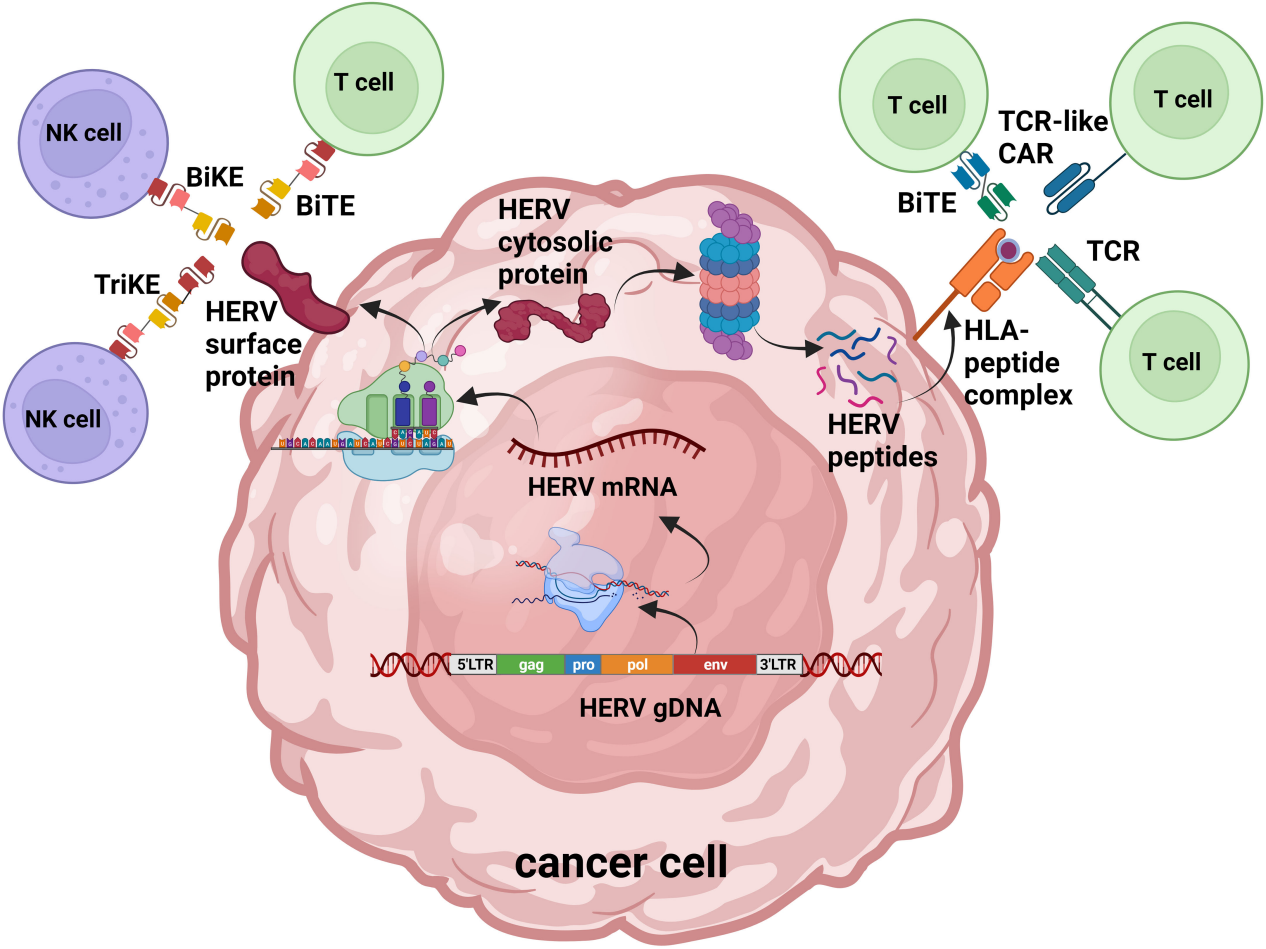
HERV protein products as antigens for cell-targeted immunotherapy. Overview of HERV-originated antigen production and presentation in cancer cells: activated HERVs are transcribed into mRNA and translated; endogenous proteins are degraded by the proteasome into peptides. HERV cell surface proteins as well as peptide fragments loaded on MHC-I can be recognized by genetically engineered T cells and NK cells. TCR, T cell receptor; CAR, chimeric antigen receptor; BiTE, bispecific T cell engager; BiKE, bispecific killer engager; TriKE, trispecific killer engager. Figure adaptation was made with BioRender.com.

While the function of HERVs in healthy tissues is largely unknown, the role they serve in cancer initiation and progression has been the focus of much research during last decades. We will review evidence showing HERVs are linked to different cancerogenic mechanisms of cell transformation and can serve as cancer-specific biomarkers and targets for cellular immunotherapy (**Figure 1**).

## Mechanisms of activation of HERVs in malignancies

Recent research in the field has demonstrated that regulation of the process of HERV activation in malignancies is more complex than previously thought. The broader role of epigenetic alterations in promoting and maintaining cancer development is now well-established (Baylin and Jones, 2011). Compared to normal tissues, the cancer epigenome exhibits dramatic changes in virtually all epigenetic control mechanisms, including mutations in chromatin-modifying enzymes and repression of tumor suppressor gene expression due to cancer-specific DNA methylation at CpG islands in their promoter regions (Baylin and Jones, 2011; Dawson and Kouzarides, 2012). Likewise, HERV elements are transcriptionally regulated through different epigenetic mechanisms (Geis and Goff, 2020).

DNA methylation is a widely studied mechanism of epigenetic regulation of HERVs that is mediated by *de novo* DNA methyltransferases (DNMTs) 3A and 3B and the maintenance methyltransferase DNMT1. These enzymes are able to methylate cytosines in previously unmethylated CpG sequences or copy pre-existing methylation marks onto new DNA strands during replication, respectively (Jin and Robertson, 2013). DNA cytosine methylation is well known mechanism inhibiting the binding of transcription factors to LTRs with about 80% of all CpGs being evolutionary methylated in repetitive elements (Iguchi-Ariga and Schaffner, 1989). Importantly, along with the original function of LTRs in retroviral integration in the host genome, LTRs that flank HERVs can play the role of alternative promoters and enhancers leading to abnormal gene expression that might contribute to tumorogenesis (Ito et al., 2017).

While the silencing of many HERV elements relies predominately on DNA methylation, other HERVs are more strongly regulated via histone modifications, such as histone methylation by heterochromatin mediators H3K9me2 and H3K9me3 (Karimi et al., 2011; Liu et al., 2018; Ohtani et al., 2018) and histone H3 protein acetylation (H3K27ac) (Chuong et al., 2013; Bannert et al., 2018). Histone methyltransferase SETDB1, which mediates chromatin compaction and epigenetic regulation of transcription in concert with heterochromatin mediators, is highly upregulated in various cancer types and was shown to regulate HERV activity as well (Fukuda and Shinkai, 2020; Lazaro-Camp et al., 2021).

Interestingly, it was found that younger HERVs are more rich in CpG content and mainly suppressed via DNA methylation whereas older HERVs shift towards silencing through histone modifications due to the evolutionary link between CpG methylation and mutation accumulation rate (Ohtani et al., 2018). Additionally, it has been shown that the level of DNA cytosine methylation inversely correlates with the expression of HERV-K proviruses, however this mechanism is not entirely responsible for their transcriptional silencing in some cell lines (Lavie et al., 2005). Older HERV-K elements have also been found to have signs of previous methylation in the form of CpG sites that have mutated to either TG or CA as a result of the methylated cytosine mutating to a thymine (Reus et al., 2001).

Besides, other epigenetic transcriptional regulators of endogenous retroviruses in mammals, Kruppel-associated box (KRAB)-containing zinc finger proteins (KZFPs) appear to be involved in the regulation of HERV transcription (Wolf et al., 2015; Bertozzi et al., 2020). Additional molecular pathways, that can mediate heritable changes in gene expression without changing genomic DNA sequence, involve post-translational modification of histone proteins and post-transcriptional gene regulation by ncRNAs (Berger et al., 2009; Ozata et al., 2019). In particular, accumulating evidence suggests that PIWI-interacting RNAs (piRNAs) are actively involved in chromatin changes, DNA methylation and protection of the genome from transposition events (Ozata et al., 2019).

Aside from incidents of epigenetic dysregulation, other mechanisms are known to activate the expression of HERVs, and in some cases to lead to progression of cancer. A topic of increasing interest is the transactivation of HERV elements by cancerogenic viruses such as Human Immunodeficiency Virus type 1 (HIV-1), Epstein-Barr virus (EBV), and Hepatitis B virus (HBV). Oncoviruses are able to regulate HERV expression via different pathways of epigenetic modifications and processing of viral transcriptional factors. For instance, HERV-K was found to be activated in HIV-1 patients via HIV-1 Tat protein involvement with NF-kB and NF-AT pathways (Gonzalez-Hernandez et al., 2012; Gonzalez-Hernandez et al., 2014). Remarkably, the discovery of cytidine deaminases (APOBEC3 proteins) and their role in the regulation of HIV-1 and other retroviral infections (Chiu and Greene, 2008) also led to uncovering their role as inhibitors of retrotransposon activity of HERV elements (Esnault et al., 2005; Lee and Bieniasz, 2007). Transcriptional activation of the HERV-K18 envelope gene that possesses superantigen activity in EBV-infected B lymphocytes was first showed by Sutkowski et al. (Sutkowski et al., 2001). Further studies revealed the pathway of this HERV activation to be through the EBV latent proteins LMP-1 and LMP-2A docking to their human cellular receptor CD21 on resting B cells (Hsiao et al., 2006; Hsiao et al., 2009). The association between HERV-K transactivation in chronic hepatitis B caused by HBV infection is still unclear and requires further studies. However, one study showed a correlation between elevated HERV-K expression levels with a worsening prognosis with hepatocellular carcinoma (Ma et al., 2016). Besides HERV-K, transactivation of the HERV-W envelope gene in human hepatoma cell line HepG2 was linked to HBV-encoded protein X (HBx) involvement with the NF-kB pathway (C. Liu et al., 2017).

Taken together, recent research showing HERV transactivation by different oncogenic viruses may provide new approaches for the treatment of some malignancies. Further exploration and a deeper understanding of the epigenetic regulation of HERVs could lead to future pharmaceutical approaches that control HERV activation and expression in different types of cancer, which potentially could be utilized in the future to augment various immunotherapy approaches. In the next part of our manuscript, we will describe the role HERVs play in a variety of tumorigenic mechanisms as well as their role in pathogenesis and immunity.

## HERV dysregulation in different types of cancer and its contribution to pathogenesis and immunity

Although most HERV elements are silenced under normal physiological conditions, re-expression of the youngest HERV family, HERV-K, has been widely detected in both hematological and solid malignancies. The first evidence came from the nucleotide hybridization where HERV-K transcripts were found in several human cell lines including Hep-2, T47D, HMT-2, KATO-III, Hela, and T47D (Ono et al., 1987). Further accumulating data validated the expression of HERV-K in multiple cancer types like breast cancer (Wang-Johanning et al., 2001; Wang-Johanning et al., 2003; Golan et al., 2008; Wang-Johanning et al., 2008; Zhao et al., 2011; Wang-Johanning et al., 2014; Tourang et al., 2021; Wei et al., 2022), human teratocarcinoma (Boller et al., 1993; Löwer et al., 1993; Boller et al., 1997; Chan et al., 2019), leukemia (Depil et al., 2002; Huang et al., 2013; Januszkiewicz-Lewandowska et al., 2013; Bergallo et al., 2017), lymphoma (Tatkiewicz et al., 2020), germ cell tumors (Herbst et al., 1996; Sauter et al., 1996; Kleiman et al., 2004; Rakoff-Nahoum et al., 2006), ovarian cancer (Armbruester et al., 2002; Wang-Johanning et al., 2007; Rycaj et al., 2015), melanoma (Schiavetti et al., 2002; Muster et al., 2003; Büscher et al., 2005; Büscher et al., 2006; Humer et al., 2006; Hahn et al., 2008; Serafino et al., 2009; Katoh et al., 2011; Schmitt et al., 2013; Krishnamurthy et al., 2015; Zhou et al., 2015), prostate cancer (Goering et al., 2011; Reis et al., 2013; Wallace et al., 2014; Goering et al., 2015), liver cancer (Ma et al., 2016), bladder cancer (Kreimer et al., 2013), lung cancer (Zare et al., 2018), kidney cancer (Weyerer et al., 2021), pancreatic cancer (Li et al., 2017), head and neck squamous cell carcinoma (Cuffel et al., 2011), soft tissue sarcoma (Giebler et al., 2018), and glioblastoma (Shah et al., 2022). In contrast to its high expression in tumors, several studies reported HERV-K to be mostly undetectable in normal tissues (Armbruester et al., 2002; Muster et al., 2003; Büscher et al., 2006; Kreimer et al., 2013; Tourang et al., 2021; Shah et al., 2022). That being said, at least one study showed elevated HERV-K HML-2 polymerase expression in healthy tissues in comparison to the matched colon cancer tissues (Dolci et al., 2020).

The HERV-K family was originally identified to have high homology to the Mouse Mammary Tumor Virus (MMTV) (Ono et al., 1986) and later was divided into ten clusters containing at least 600 proviruses (named HML1-10) (Vargiu et al., 2016). Because multiple genomes of these youngest HERVs possess copies of intact ORFs that are closely related, it is sometimes difficult to perform accurate HERV-K loci genomic location mapping (Wildschutte et al., 2016). Importantly, a recent analysis of 1000 Genomes Project Data revealed insertional polymorphisms of HERV-K elements in humans, at both an individual as well as global population level (W. Li et al., 2019). This comprehensive computational analysis demonstrated striking differences in the prevalence of HERV-K combinations between Europeans, Africans and East Asians, highlighting that HERV-K provirus polymorphisms occur at a greater frequency than was previously appreciated. Relatively “recent” HERV-K incorporation and retrotransposition could potentially account for genome instability and cellular processes distraction.

HERV-K transactivation in breast cancer has been observed and actively studied by a number of different groups. Analysis of combining RNA sequencing (RNA-Seq) datasets from nine laboratories comprising tumor samples from breast cancer, normal tissues adjacent to tumors, and breast tissue controls from healthy donors revealed overexpression of several HERV-K elements in tumor tissues with expression levels being highly heterogeneous amongst different breast cancer histological variants (Wei et al., 2022). The finding of HERV-K overexpression in tumors *vs* normal breast tissues suggests its potential use as a biomarker for breast cancer. Other recent research highlighted an association between HERV-K expression levels and tumor size, TNM stage, and lymph node metastasis, with HERV-K expression in tumors being associated with poor overall survival (Zhao et al., 2011). The same correlation was also identified in patients with liver cancer, where expression levels of HERV-K were associated with cirrhosis, tumor differentiation and TNM stage (Ma et al., 2016). Further, a recent study showed overexpression of several HERV-K transcripts in glioma cell lines compared to normal human astrocyte cell lines. Notably, the highest glioma expression relative to astrocyte controls of the HERV-K locus HML-6 (on chr. 19q encoding the small Env protein ERVK3-1) was observed in the most highly proliferative glioma cell lines (Shah et al., 2022). Remarkably, a bioinformatic analysis of different datasets revealed that increased expression of HML-6 in the tumors of glioblastoma patients was linked to reduced survival (Shah et al., 2022).

Correlating with high proviral gene expression, antibodies against HERV-K-originated protein products have been detected at high titers in the sera of patients with various tumors. For example, HERV-K Env antibodies have been detected at high levels in the sera of patients with seminomas and mixed germ cell tumors (Sauter et al., 1996; Boller et al., 1997). Similarly, patients with melanoma have been observed to have a high prevalence of antibodies against HERV-K envelope epitopes that appear to be stage dependent (Humer et al., 2006). Additionally, anti-HERV-K Env and Gag antibodies were observed to be elevated in patients with breast cancer (Wang-Johanning et al., 2008; Wang-Johanning et al., 2014), prostate cancer (Reis et al., 2013; Manca et al., 2022), pancreatic cancer (Li et al., 2017), ovarian cancer (Wang-Johanning et al., 2007), with changes in the titer of anti-HERV-K antibodies being associated with the disease course. In patients with germ cell tumors, disease remission was accompanied by a decrease in anti-HERV-K antibody titers while progression of disease and disease relapse correlated with an increase or stable antibody titers (Kleiman et al., 2004). Likewise, a study of HERV-K Gag protein expression in patients with prostate cancer revealed a correlation between antibody levels and disease stage (Reis et al., 2013). Remarkably, results of a blind study of sera collected from melanoma patients at different disease stages suggested anti-HERV-K Gag and Env antibody titers could be used as prognostic markers of disease (Hahn et al., 2008). In particular, a statistical analysis of anti-HERV-K antibody reactivity compared to other known prognostic indicators of melanoma outcome demonstrated a correlation between increased antibody titers with reduced survival in patients with early stage disease.

A potentially important role of HERV-K in tumorigenesis or cancer progression has been shown for some types of cancer. For example, one study evaluating the biological impact of HERV-K expression in breast cancer cells demonstrated shRNA knockdown of the HERV-K envelope impacted cell proliferation, migration and invasion (Zhou et al., 2016). Additionally, bioinformatics analysis of RNA-seq data conducted on these experiments showed a link between HERV-K expression and p53, Myc, and Ras signaling pathways. Similarly, shRNA knockdown of HERV-K envelope expression in pancreatic cancer cell lines downregulated RAS-ERK-RSK, a signaling pathway known to be important in pancreatic cancer progression (Li et al., 2017). Furthermore, several studies provide data implicating the HERV-K proteins Rec and Np9, products of alternative envelope splicing, as being potential oncogenes in a variety of cancers through their interaction with functionally important cellular proteins (Armbruester et al., 2002; Armbruester et al., 2004; Büscher et al., 2005; Büscher et al., 2006; Denne et al., 2007; Katoh et al., 2011; Huang et al., 2013; Fischer et al., 2014; Schmitt et al., 2015; Chan et al., 2019). Further research elucidating the full biological sequalae of expression of this HERV subfamily could lead to future therapeutic applications.

In addition to HERV-K, activation of other HERV families like HERV-W, HERV-H, and HERV-E has also been described in a variety of different types of tumors and remains an active area of investigation by a growing number of groups. For example, six HERV-W loci were found to be overexpressed in testicular cancer, including ERVWE1 which was previously thought to have placenta-restricted expression (Gimenez et al., 2010). Other studies have demonstrated upregulation of envelope spliced transcripts from different HERV-H loci in colon cancer cell lines (Liang et al., 2012) as well as in tumors from patients with head and neck cancer (Kolbe et al., 2020). Interestingly, concurrent expression of different HERV groups was found in ovarian cancer cells (such as ERV-K, ERV-E, and ERV3) (Wang-Johanning et al., 2007) as well as in kidney cancer (such as ERV-K, ERV-H, and ERV-E) (Weyerer et al., 2021). Additionally, the most recent bioinformatical analysis of the Cancer Genome Atlas (TCGA) datasets evaluating HERV contribution in cancer pathogenesis revealed specific expression signatures of different HERVs in uveal melanoma (UM) (Bendall et al., 2022). In particular, analysis of RNA-seq data from 80 primary UM samples identified the expression of 17 HERV loci (including HERVE_Xp11.23 with coding potential). Remarkably, the landscape of HERV expression could be used to classify UM tumors into different subtypes with high accuracy, with this classification correlating well with subtypes identified using other molecular signatures (Bendall et al., 2022). A growing number of studies characterizing HERV-E expression in kidney cancer have been reported over the last few years. Our group discovered expression of a unique HERV-E loci located on chr. 6q15 (named CT-RCC HERV-E (Takahashi et al., 2008) or ERVE-4 according to HGNC nomenclature) in renal cell carcinoma (RCC). HERV-E expression was identified following the isolation of a CTL clone with specific cytotoxicity against kidney tumor cells from a patient with metastatic ccRCC following an allogeneic stem cell transplant (Takahashi et al., 2008). We found this HERV element to be specifically expressed in the most common clear cell type of RCC (ccRCC), with no expression detected in normal tissues or other cancers (Takahashi et al., 2008; Cherkasova et al., 2016). Subsequently, we discovered select CT-RCC HERV-E expression in ccRCC was regulated by transcription factor HIF-2α (known to be upregulated by Von Hippel-Lindau (VHL) tumor suppressor inactivation in ccRCC) and occurred only when the proviral 5’LTR was demethylated (Cherkasova et al., 2011). Remarkably, using bioinformatic analysis of RNA-Seq data in TCGA, exclusive expression of this HERV-E loci was found by Rooney et al. to be associated with elevated levels of cytolytic activity in ccRCC (Rooney et al., 2015). Furthermore, a recent genomic and transcriptomic analyses performed by Ficial et al. in tumor tissues from ccRCC patients treated with the anti-PD-1 antibody nivolumab demonstrated that the higher CT-RCC HERV-E (ERVE-4) expression was associated with an increased durable response rate (DRR) and a significantly longer progression-free survival (PFS) (Ficial et al., 2021). This finding highlighted for the first time that HERV-E element activation could potentially serve as a biomarker predicting response to checkpoint blockade immunotherapy. Additionally, a bioinformatic study conducted by a different group using TCGA data from kidney cancer patients treated with immunotherapy identified expression of a different HERV-E loci (ERVE 4700) with higher levels of expression in ccRCC compared to normal kidney tissue where expression levels were only moderate (Smith et al., 2018). However, in contrast to CT-RCC HERV-E which appears to have prognostic value for predicting response to checkpoint inhibitors, Ficial et al. did not observe any association between ERVE 4700 expression and DRR and PFS in patients treated with nivolumab (Ficial et al., 2021).

Besides potential clinical prognostic value, it has also been shown that HERV can contribute to the IFN regulatory network and stimulate IFN-induced responses (Chuong et al., 2016). Chuong et al. found 20 HERVs containing STAT1 (the primary transcription factor activated by IFNs) binding motifs in their LTRs and demonstrated their role as promoters of certain IFN-stimulated genes (Chuong et al., 2016). In particular, they showed that MER41 (HERVW9) deletion from human genome by Crispr-Cas9 impaired the expression of adjacent IFNG-induced genes. A research into the mechanism of action of DNA methylation inhibitors in colorectal cancer identified HERV elements to function as a source of endogenous double-stranded RNAs (dsRNA) that triggered viral immune recognition and IFN responses (Roulois et al., 2015; Attermann et al., 2018). Remarkably, exposure of colorectal cancer cells to 5-aza-2-deoxycydine treatment was shown to lead to an anti-proliferative response mediated by “viral mimicry” brought on by demethylation and transcription of HERVs. Additional wet and *ex vivo* research, as well as correlational studies using clinical data from cancer patients is needed to fully understand HERV signatures and the complex roles they play in various solid and hematological malignancies.

## HERV-originated products as potential targets for cancer immunotherapy

The findings of anti-HERV antibodies in sera of cancer patients suggest that HERV products can be used as targets in cancer immunotherapy. For instance, Wang-Johanning et al. discovered the monoclonal anti-HERV-K Env antibody 6H5, which inhibited the proliferation of breast cancer cells both *in vitro* and *in vivo* (Wang-Johanning et al., 2012). Subsequently, 6H5 single-chain fragment variable (scFv) sequence was used to generate a chimeric antigen receptor (CAR) to be expressed in T cells (Zhou et al., 2015). It was shown that when 6H5 CAR T cells were co-cultured with HERV-K-positive breast cancer cells, they released IFN-γ, TNF-α, IL-2 and killed the tumor cells. Moreover, the ability of 6H5 CAR T cells to kill primary tumors and reduce metastases in various organs in tumor bearing mice suggests these cells could be of therapeutic value. Because HERV-K was found to be highly expressed in melanoma, 6H5 CAR T cells were also tested against melanoma cells (Krishnamurthy et al., 2015). 6H5 CAR T cells showed specific killing of HERV-K-positive melanoma cell lines with killing decreasing significantly when HERV-K *env* gene expression in melanoma cells was knocked down. Similarly to breast cancer bearing mice, an anti-tumor effect of 6H5 CAR T cells was demonstrated in a melanoma xenograft model.

Peptides derived from HERVs also appear to be attractive candidates for tumor targeted cellular immunotherapy against a number of different cancer types including breast cancer (Wang-Johanning et al., 2008), colorectal cancer (Mullins and Linnebacher, 2012), kidney cancer (Takahashi et al., 2008; Cherkasova et al., 2016), melanoma (Schiavetti et al., 2002; Krone et al., 2005), and seminoma (Rakoff-Nahoum et al., 2006). Schiavetti et al. discovered a unique CTL clone (CTL13) which killed melanoma tumor cells in an HLA-A02-dependent manner. It was found that the peptide recognized by CTL13 clone was translated from a short ORF of HERV-K-MEL provirus located on chr. 16 (Schiavetti et al., 2002). Mullins et al. used HLA-A02-restricted antigenic peptides predicted to be derived from a Gag protein of HERV-H Xp22.3 that is overexpressed in colorectal cancer to stimulate peptide-specific T cells. Remarkably, CTLs were generated with that specifically recognized and killed HERV-H Xp22.3-positive/HLA-A02-positive colorectal cancer cells (Mullins and Linnebacher, 2012). Finally, Rycaj et al. demonstrated that CTL with tumor specificity for autologous patient ovarian cancer cells could be generated by pulsing dendritic cells with HERV-K Env antigens (Rycaj et al., 2015).

To have the greatest utility for targeted immunotherapy and to reduce the risk of off-tumor toxicity, it is important to identify and target HERV-derived antigens that have selective expression in cancer. The CT-RCC HERV-E identified by our group to have selective expression in most clear cell renal cell carcinomas seems to foot this bill. Our group isolated a CD8+ T cell clone that had tumor specific killing of kidney cancer cells from a patient who had durable regression of metastatic ccRCC following an allogeneic stem cell transplant. The T cell receptor (TCR) from this clone recognized an HLA-A11-restricted 10mer peptide named CT-RCC-1 that was shown to be encoded by the CT-RCC HERV-E (Takahashi et al., 2008). When T cells from the PBMC from healthy donors were transduced with a retroviral vector encoding the genes from this TCR, they acquired the ability to selectively kill HLA-A11^+^/CT-RCC HERV-E expressing ccRCC tumors *in vitro* and *in vivo* in a murine xenograft tumor model (Barisic et al., 2023). Based on these preclinical data, we initiated the first ever trial in humans exploring the potential of T cell immunotherapy targeting a HERV antigen to treat cancer. In this ongoing phase I trial being conducted by the NHLBI at the NIH Clinical Center, escalating doses of autologous CD8+ T cells transduced to express a TCR recognizing the CT-RCC HERV-E antigen are infused into HLA-A11 positive patients with metastatic ccRCC in a dose escalating fashion (NCT03354390). Although the TCR being evaluated in this trial is highly cytotoxic *in vitro* and *in vivo* to ccRCC cells, only a minority of patients express HLA-A11 which is required for recognition of the HERV-E derived CT-RCC-1 peptide. Therefore, identifying and subsequently targeting HERV-E-derived peptides that are presented by HLA Class I molecules more commonly expressed in humans remains an active research focus of our group. Previously, we identified a transcript encoding the HERV-E Env gene which was found to have partially preserved ORFs in SU and TM domains. Remarkably, we found HLA-A02-restricted peptides encoded by these transcripts that could stimulate CTL *in vitro* to kill ccRCC cells in an HLA-A02 dependent fashion (Cherkasova et al., 2016). Our most recent efforts have focused on identifying scFv fragments that recognize HERV-E derived peptides that are presented in the context of commonly expressed HLA molecules (Myrio Tx) (CAR TCR Summit: Myrio Tx Shares Transformational Approach to Targeted Human Antibody-Based Therapies) as well as monoclonal antibodies that bind HERV-E derived proteins that are surface expressed on ccRCC cells (Adagene) (Adagene, 2021). The scFv regions from these binders and/or mAbs could be used to generate CAR T cells as well as bispecific antibodies (bsAbs) as an off the shelf therapeutic to treat ccRCC. BsAbs, because of their ability to engage two different targets, are being intensively investigated in targeted cancer immunotherapies and are of great clinical interest and potential. The bispecific T cell engager (BiTE) therapy, using constructs linking a T cell to a tumor cell via anti-CD3 and anti-tumor scFvs, constitutes a promising approach for treatment of a broader range of hematological malignancies and a variety of different types of solid tumors (Goebeler and Bargou, 2020; Voynov et al., 2020; Sheridan, 2021; Zhou et al., 2021). Generation of bispecific NK cell engagers (BiKEs), created from the fusion of anti-CD16 scFv and anti-tumor scFv, or tri-specific NK cell engagers (TriKEs), carrying combinations of two different anti-tumor scFvs, for the direct targeting of genetically modify NK cells to cancer cells have also attracted wide attention as a promising new anticancer therapeutics (Tay et al., 2016). Multiple BiKEs and TriKEs have been shown to have cytotoxic activity against various tumor antigens are currently in preclinical studies and drug development (Allen et al., 2021; Liu et al., 2021; Huan et al., 2023).

Remarkably, two studies examined the potential of vaccination against HERV proteins as a possible way to prime the immune system to generate anti-tumor responses (Kraus et al., 2013; Kraus et al., 2014). Kraus et al. generated recombinant vaccinia viruses Ankara (MVA-HKenv and MVA-HKcon) expressing HERV-K Env and Gag proteins, respectively. Notably, a single vaccination with these vaccinia viruses in mice bearing murine renal carcinoma (Renca) cells that had been modified to express these HERV-K proteins significantly reduced the size and number of tumor nodules compared to vaccination with unmodified MVA (Kraus et al., 2013; Kraus et al., 2014). These data demonstrated for the first time that HERV proteins can be used for vaccine development as an alternative immunotherapy strategy to treat cancer.

## Conclusions

This review examines the data accumulated over the past decades on the growing understanding of HERV dysregulation in various tumors and their contribution to cancer pathogenesis. There is increasing evidence that HERV-derived antigens can serve as viable targets for cellular immunotherapy and can also be used as diagnostic and prognostic biomarkers. There are various strategies used in modern oncology for cellular therapy, including mAbs, bispecific and trispecific antibodies, and modified T cells and NK cells armed with TCRs and CARs to target peptide-MHC complexes or intact proteins present on the tumor surface (**Figure 1**). There is growing data demonstrating that HERVs can be activated in a variety of different tumors and can serve as therapeutic targets in the rapidly expanding field of tumor immunotherapy. As mentioned previously, a requirement for tumor immuno-therapists will be to identify and subsequently target unique HERV-derived antigens that have tumor-restricted expression to reduce the risk of off-tumor toxicity.

Although recent studies shown different mechanisms accounting for HERV activation in specific cancers and an association between expression of HERV antigens and anti-tumor immune responses, most evidence supporting the value of HERV targeted immunotherapy has come from mouse models. Clinical therapeutics targeting HERV-derived antigens are only now just beginning to be pursued in humans. Further studies are needed to shed light on the exact molecular mechanisms and pathways involved in regulating HERV expression and the specific immune responses that occur against HERV antigens in cancer and other diseases. Improved sequencing and genome mapping methods might enhance future understanding of the cancer-specific HERV landscape, which could lead to new therapeutic strategies for targeting both solid tumors and hematological malignancies. More research is also needed to identify specific HERV elements that may be involved in cancer pathogenesis and elucidate the corresponding molecular mechanisms with the applicability of this knowledge to development of new therapeutic approaches. Finally, given the importance of antigen expression levels impacting the effectiveness of cancer immunotherapy, it will be important to characterize molecular as well as pharmaceutical strategies to regulate HERV epigenetic and transcriptional activation in cancer cells. A better understanding of the complex interplay between HERV immunogenic burden, cancer-specific molecular pathways regulating HERV expression, and HERV-antigen reactive immune infiltration in advanced malignancies will facilitate the development of novel HERV-based cancer diagnostics and therapeutics.

## Author contributions

EC: Conceptualization, Writing – original draft. LC: Writing – original draft. RC: Writing – original draft, Conceptualization, Supervision.
